# Challenges related to self-assessment of active ageing during the Covid-19 pandemic in Sweden

**DOI:** 10.1186/s13104-022-06059-3

**Published:** 2022-05-13

**Authors:** Magnus Zingmark, Frida Nordeström, Susanne Iwarsson

**Affiliations:** 1 Department of Health Sciences, Lund University, P.O. Box 157, 221 00 Lund, Sweden; 2 Health and Social Care Administration, Municipality of Östersund, Östersund, Sweden

**Keywords:** Architectural Accessibility, Activities of Daily Living, Housing, Aging in Place, Mobility, Neighborhood Characteristics, Social participation

## Abstract

**Objective:**

The aim is to describe and reflect upon potentially pandemic-related impact on self-assessments of active ageing. As part of the baseline data collection in the Prospective RELOC-AGE (ClinicalTrials.gov NCT04765696) study, telephone interviews, including the University of Jyvaskyla Active Aging Scale (UJACAS) were conducted with 820 people 55 years or older listed with an interest of relocation at three housing companies in Sweden. Field notes alongside the interviews focused on two topics: (1) how respondents reasoned and replied to the questions included in the UJACAS; (2) whether there were specific items that seemed to be affected by the pandemic.

**Results:**

For four items (Participating in events, Exercising, Maintaining friendships, Getting to know new people), recurrent comments indicated that respondents had been affected by the pandemic situation regarding one or more of the facets in UJACAS: will to act, ability to act, opportunity to act, or frequency or extent of doing the activity. Opportunities to act was most frequently commented on as a factor affected by restricted participation in activities. As Prospective RELOC-AGE is a longitudinal study focused on associations between housing, relocation and active ageing, it is imperative to consider the potential pandemic-related impact on baseline data in future analyses.

## Introduction

Prospective RELOC-AGE is a longitudinal mixed-methods study exploring associations of housing, relocation, and active and healthy ageing in Sweden. Based on the hypothesis that housing choices and relocation are associated with active and healthy ageing, one facet of the data collection includes an individual self-assessment of active ageing. Prospective RELOC-AGE is to the best of our knowledge the first study on housing choices and relocation among older people in which active ageing is used as a core perspective and outcome.

Active ageing can be seen as a goal for policies referring to “the process of optimizing opportunities for health and participation in the society for all people in line with their needs, goals and capacities as they age” [1]. Initiatives to promote active aging can be seen from a societal perspective in terms of providing accessible environments including transportation and housing, or from a service provider perspective, for example, by implementation of health-promoting interventions. Active ageing can also be seen from an individual perspective, in terms of strategies and behaviours that the individual can adopt to optimize his/her opportunities for participation and health. On the individual level, active ageing has been described as striving for well-being through activity as per one’s goals, opportunities, and abilities [2]. One prerequisite for active ageing in terms of environmental opportunities or hindrances is therefore housing and services that are tailored to support independent living, for example, by age-friendly housing or relocation [3].

The target population for Prospective RELOC-AGE are people 55 years or older (i.e., born in 1966 or earlier) and listed as interested of relocation at three housing companies in Sweden [4]. The data collection began in March 2021, which coincided with the onset of the Coronavirus Disease of 2019 (COVID-19) pandemic in Sweden. While no extensive lock-down was implemented in Sweden, pandemic-related restrictions and strict recommendations particularly impacted people aged 70 years and older. As Prospective RELOC-AGE is a longitudinal study including older adults, to inform forthcoming data collection waves the validity of the baseline data must be considered in the light of the potentially abnormal patterns of engagement in daily activities. Accordingly, the aim of this research note is to describe the potentially pandemic-related impact on self-assessments of active ageing.

## Main text

### Methods

The full scope of the study design in Prospective RELOC-AGE is available at ClinicalTrials.gov NCT04765696 and in a study protocol [4]. During the first step of the data collection (a web-based survey), respondents (n = 1964) were asked if they were willing to participate in an additional telephone interview. The telephone interview included two self-assessments of perceived aspects of housing [5]. These assessments were conducted by telephone since previous studies had indicated threats to the validity because of challenges for respondents to apprehend the underlying concepts. As we had no previous experiences of assessments of active ageing, we included this assessment in the telephone interview as well.

Active ageing was self-assessed with the University of Jyvaskyla Active Aging Scale (UJACAS) [2]. Before the telephone interview, a paper version of the questionnaire was sent to the respondents for them to prepare and thereby facilitate the recording of responses. The UJACAS contained 17 items ranked for four facets of active aging (will to act, ability to act, opportunity to act, and frequency/extent of doing the activity) on a five-point Likert scale ranging from not at all/very low to very much/very high (Table 1). Respondents were asked to consider the previous four weeks when scoring. Together the items capture a single construct reflecting individual active ageing (total score range = 0–272).

**Table 1:** Items included in University of Jyvaskyla Active Aging Scale (UJACAS), and items for which responses were potentially affected by pandemic-related restrictions

UJACAS item^a^	Responses affected
Practicing memory	
Using a computer or pad	
Advancing matters in one’s own life	
Exercising	X
Enjoying the outdoors	
Taking care of one’s personal appearance	
Crafting or “do it yourself” (DIY) activities	
Making one’s home cosy and pleasant	
Helping others	
Maintaining friendships	X
Getting to know new people	X
Balancing personal finances	
Making one’s days interesting	
Practicing artistic hobbies	
Participating in events	X
Advancing societal/communal matters	
Doing things in accordance with one’s world view	

^a^For each item respondents ranked their will to act, ability to act, opportunity to act, and frequency or extent of doing the activity, on a five-point Likert scale ranging from not at all/very low to very much/very high

In all, 1412 survey respondents (72%) agreed to be contacted for a telephone interview based on consecutive recruitment. A paper version of the UJACAS was sent to 1011 persons, followed by attempts to contact all of them by telephone to confirm time for an interview lasting 15–45 min. Interviews were completed with N = 820 (response rate 81%; 51% women). The mean age was 69.7 years (SD 7.65; range 54–92 years).

The staff involved in administration of the telephone interviews kept field notes focused on two topics: (1) how respondents reasoned and replied to the questions included in UJACAS; (2) whether there were specific items that seemed to be affected by the COVID-19 pandemic, including related recommendations and restrictions. These topics generated field notes related to 81 of the interviews. After 200 interviews saturation was reached and taking field notes was terminated.

## Results

For four items in UJACAS there were recurrent comments that indicated that respondents had been affected by the pandemic situation regarding one or more of the facets: will to act, ability to act, opportunity to act, or frequency or extent of doing the activity (Table 1).

### Participating in events

Because many events were cancelled due to pandemic-related restrictions, the responses to the item *participating in events* had been affected. One respondent commented:

“I have been engaged a lot in organizations but now I just can´t.”

To some extent, participating in online events was mentioned as an alternative providing new opportunities to participate in events that otherwise had been difficult to attend, for example, due to the need to travel. One respondent who lived far from the capital had positive experiences:

“There are so many events to participate in, everything is digital, I can join events in Stockholm now, I never could do that before!”

In contrast to such positive experiences, others expressed the lack of social contacts leading to less motivation to participate, or that too many digital events were experienced as strenuous:

“It is so exhausting with everything online, I can´t stand yet another online meeting.”

### Exercising

The item *exercising* was discussed by several participants in terms of that there was no clear-cut line between strenuous activity and less demanding activity. Overall, the responses to this item did not indicate that respondents had reduced their engagement in exercise but rather changed the type of exercise they did:

“I haven´t been able to go to water aerobics, instead I have done other activities such as going for walks“.

### Maintaining friendships

In relation to the item *maintaining friendships* comments from the respondents indicated that there were differences both regarding whether a person worked or not and whether he/she cohabited or lived alone. For those working, the need for social relations was to some extent met despite the pandemic situation, and the energy to engage in further social contacts outside working hours was limited. In contrast, those who did not work expressed that they needed to actively engage to maintain friendships and social contacts.

“It is difficult now, but we call each other and meet in the garden.”

“We have met every other week, but we kept a distance and could see each other.”

### Getting to know new people

For the item *getting to know new people* respondents expressed lack of motivation as well as restricted opportunities due to the pandemic.

“I just don’t want it right now. It is not like I use to be but I just haven´t had the surge during the pandemic. Even if I had wanted, there has been a lack of opportunities.”

Once again, whether the respondent worked or not seemed to have an impact on the responses to this item:

“I don´t have that kind of social need privately. As part of my work, I look for new contacts. I don´t have the energy or motivation to do that in my leisure time as well.”

### Item responses not affected

For 13 of the 17 UJACAS items, there were no comments that indicated that the will to act, ability to act, opportunity to act, or frequency or extent of doing the activity had been affected by the pandemic situation (Table 1).

### Differentiating between facets of active ageing

Several respondents had difficulties to differentiate between the ability and opportunity to act. After clarifications from the administrators, their replies clearly indicated that the ability to act was not affected by pandemic-related restrictions whereas opportunities to act were. However, as described by some respondents, low mood and anxiety associated with a fear of being lonely or being infected by COVID-19 resulted in a restricted ability to act. As described above, the will to act was reduced specifically for the item getting to know new people.

## Discussion

The results indicate a COVID-19 pandemic-related impact on four of the 17 items in UJACAS during the baseline data collection for Prospective RELOC-AGE [4], in particular regarding opportunities to act. In Sweden the pandemic-related restrictions and recommendations impacted on people of all ages, but especially those 70 years or older were constantly addressed as a vulnerable group [6]. With a sample mean age of 69 the results indicating that active ageing was affected are not surprising, and are in line with Siltanen et al. who concluded that a decline in active ageing mainly is due to opportunities to live an active life [7]. In line with other studies [8, 9], our results indicate that older adults did engage in exercise even though the specific activities and locations were different. Whereas mobility limitations seem to be associated with a larger decline in active ageing, a high level of self-resilience has been found to be protective [7]. Further, older adults with high levels of COVID-19-related health literacy were more likely to perceive the recommendations had restricted their daily lives [10].

The pandemic-related restrictions are a period effect, which likely have affected older adults’ opportunities to live an active life. On the one hand, our results indicate that the UJACAS validly can capture such circumstances. On the other hand, because the instructions for the self-assessment was that the respondent should consider only the previous four weeks when scoring, the instrument is sensitive to a current situation and does not capture the overall individual level of active ageing. That is, an individual who under normal circumstances is very active, for example, regarding participation in events might appear as inactive in the baseline data of the Prospective RELOC-AGE study. This is an important observation, which must be considered in any longitudinal study targeting aspects of activity and active ageing during the pandemic period. To what extent these circumstances affected active ageing or other health-related outcomes in our cohort remains to be explored as we proceed further into the analysis phase. For example, it will be important to investigate whether there were differences between age groups or in relation to other individual baseline characteristics as regards active ageing. The upcoming analyses of the complete baseline data will add knowledge on such associations. Most important, the period effects must be taken into serious consideration when interpreting the longitudinal results of the study.

## Limitations

It might be a limitation that field notes were taken only during 10% of the telephone interviews. However, the administrators continued taking field notes until saturation was reached. Nevertheless, because numerous participants had a need to discuss how to interpret items and the four different aspects of active ageing included in UJACAS, there might be threats to reliability and overall validity that require further attention. The telephone interview administration mode was chosen to counteract such threats, but an ongoing psychometric study will shed light on such matters.

## Data Availability

Data collected for this research note will, after de-identification, be available on reasonable request after publication in peer reviewed journals; the prerequisite for this is a data transfer agreement, approved by legal departments of the institutions of both the requesting researcher and the researchers that provided data for the study. Proposals should be directed to: magnus.zingmark@med.lu.se.

## Abbreviations

COVID-19: Coronavirus Disease of 2019
UJACAS: University of Jyvaskyla Active Aging Scale
